# Use of bioelectrical impedance analysis in centenarians: a systematic review

**DOI:** 10.1007/s40520-022-02282-x

**Published:** 2022-10-26

**Authors:** Caterina Mandalà, Nicola Veronese, Ligia J. Dominguez, Giuseppina Candore, Giulia Accardi, Lee Smith, Maria Trinidad Herrero, Mario Barbagallo

**Affiliations:** 1 Geriatric Unit, Department of Medicine, University of Palermo, Via del Vespro 141, 90127 Palermo, Italy; 2 School of Medicine and Surgery, University of Enna “Kore”, 94100 Enna, Italy; 3 Laboratory of Immunopathology and Immunosenescence, Department of Biomedicine, Neurosciences and Advanced Diagnostics, University of Palermo, Palermo, Italy; 4 Centre for Health, Performance and Wellbeing, Anglia Ruskin University, Cambridge, UK; 5 Clinical and Experimental Neuroscience (NiCE-IMIB). School of Medicine, Department of Human Anatomy and Psychobiology, Institute for Aging Research, Universidad de Murcia, Campus Mare Nostrum, Murcia, Spain

**Keywords:** Body composition, Fat mass, Fat-free mass, Obesity, Hydration, Centenarian

## Abstract

**Background:**

Centenarians often represent one of the best examples of aging successfully. However, the role of body composition or hydration status assessed with bioelectrical impedance analysis (BIA) is poorly explored in this population. Therefore, the aim of this systematic review was to better understand the use and the role of BIA for evaluating body composition and hydration status in centenarians.

**Methods:**

We conducted a systematic review of the literature up to the 1st of May, 2022 for published articles providing data on BIA to evaluate body composition parameters or hydration status in centenarians. Data were summarized descriptively because a meta-analysis was not possible due to the scarcity of available studies.

**Results:**

Among 2222 articles screened, four were eligible including 291 centenarians (mean age: 100.5 years) who were mainly women (88%). In one study, BIA overestimated fat-free mass and underestimated fat mass when compared to deuterium oxide dilution. Another study carried out in Italy including 14 centenarians found a significant correlation between BIA and fat-free mass evaluated using anthropometric tools. In one study, BIA showed a significant agreement with anthropometric measures of fat mass. In the same sample, sarcopenia and dehydration, evaluated with BIA, had a high prevalence.

**Conclusion:**

BIA may be used for assessing body composition in centenarians, but research is limited to a few studies suggesting the need of future research in this area.

## Introduction

Identification of correct research methods is crucial in order to achieve diagnostic accuracy and high predictive ability in clinical measures to guide care strategies in older people, including centenarians [1]. Epidemiological studies have reported that the number of centenarians exponentially increased worldwide throughout the twenty-first century [2, 3]. In 2015, there were approximately 417,000 individuals aged 100 or more years worldwide; according to the Population Division of the United Nations this figure will likely exceed over 20 million people in 2100 [2, 3].

Aging is characterized by several progressive changes, which are associated with an increased susceptibility and vulnerability to the development of disease and associated consequences [1, 4]. This has recently introduced the concept of senescence of the whole body [5, 6].

Hormonal alterations, poor nutritional status, dysregulation of the neuromuscular system, and an increased pro-inflammatory environment may promote changes in the body composition of older people [1, 4]. However, the loss of muscle and fat-free mass is not linear although it does accelerate with increasing age [7]. Moreover, total body water [8] and the metabolism rate significantly decrease with age, in proportion with the decreasing of metabolic active mass, i.e., fat-free mass [4, 9].

Changes in body composition that occur during aging not only lead to a reduction in muscle mass but in parallel there is an increased adiposity and modifications in its body distribution [10, 11]. In addition, there is an age-dependent increase in the accumulation of lipid droplets as visceral, muscle and liver fat with an opposite tendency for subcutaneous fat mass [12]. These modifications in body composition are recognized risk factors for various age-associated diseases, such as type 2 diabetes and cardiovascular disease and their related complications [13]. Centenarians often have fewer chronic diseases than non-centenarians and before the age of 85 tend to show slower health changes [14].

Currently, there are several methods for estimating body fat such as air displacement plethysmography, dual-energy X-ray absorptiometry, and magnetic resonance imaging, among others [15]. However, they are expensive, complex, implicate radiation risk, and are problematic to access as the equipment requires dedicated space and are not mobile, rendering their use challenging in the usual clinical settings [16, 17]. These tools have been used for clinical assessment of body composition in geriatric medicine [1, 18].

Bioelectrical impedance analysis (BIA) is a widely used tool to assess body composition, which is a portable, noninvasive, and inexpensive method [19, 20]. The use of BIA does not involve substantial ionizing radiation, has limited costs, can be performed in practically every person, and has minimal between-observer variations. BIA has been validated in healthy populations as well as in those with chronic diseases with a validated BIA equation, appropriate regarding to age, sex and ethnicity [21]. BIA allows the measurement of body composition and hydration status outcomes using only two parameters, i.e., resistance and reactance [19, 20]. Therefore, BIA may be applied across different settings, populations, and medical conditions [19, 20, 22].

However, among those with extreme body mass index (BMI) ranges and with abnormal hydration, the clinical use of BIA cannot be recommended for routine assessment until further validation has been performed in these conditions. Multi-frequency and segmental-BIA may have advantages over single frequency-BIA for evaluating people with severe obesity or altered hydration; nevertheless, further validation is still necessary [19–22]. In other conditions characterized by an altered fluid composition, e.g., heart failure, this method has limitations as well [15]. An advantage is that some BIA devices allow home evaluation. In a trial of healthy adults, aged 21–60 years with BMI ranging 18.6–40.5 kg/m^2^, body composition was measured with dual X-ray absorptiometry and compared to regional home-assessed tetrapolar BIA plethysmography with surface electrode placements on the upper and lower limbs. However, the use of regional impedance devices to assess body fatness is often limited by poor precision and accuracy [23].

Nevertheless, BIA analysis may be useful to estimate the composition of different body compartments in centenarians, often representing one of the best examples of aging successfully and poorly studied in terms of body composition and hydration status.

Given this background, the aim of the present systematic review was to collate and synthesize literature on the use of BIA in centenarians and evaluate its role in the assessment of body composition and hydration status in centenarians.

## Materials and methods

This systematic review adhered to the Preferred Reporting Items for Systematic Reviews and Meta-Analyses (PRISMA) statement [24] and followed a pre-planned, but unpublished protocol available by contacting the corresponding author.

### Data sources and searches

Two investigators (NV and CM) independently conducted a literature search using Pubmed/Medline, Embase, Web of Science, and Scopus, from database inception to 01st May 2022. In PubMed, the following search strategy was used: (“Electric Impedance” [MeSH Terms] OR “impedance electric” [All Fields] OR “Electrical Impedance” [All Fields] OR “impedance electrical” [All Fields] OR (“Electric Impedance” [MeSH Terms] OR (“electric” [All Fields] AND “impedance” [All Fields]) OR “Electric Impedance” [All Fields] OR “impedance” [All Fields] OR “impedances” [All Fields] OR “impedance” [All Fields] OR “impedivity” [All Fields]) OR “Electric Resistance” [All Fields] OR “resistance electric” [All Fields] OR “Electrical Resistance” [All Fields] OR “resistance electrical” [All Fields] OR “Ohmic Resistance” [All Fields] OR “Ohmic Resistances” [All Fields] OR “resistance ohmic” [All Fields] OR “Bioelectrical Impedance” [All Fields] OR “Bioelectric Impedance” [All Fields]) AND (80andover[Filter]). The search was then adapted to the other databases. Reference lists of included articles were hand searched to identify any potential additional relevant articles. Any inconsistencies were resolved by consensus, with a senior author (LS).

### Study selection

Inclusion criteria for this systematic review were as follows: (i) centenarians; (ii) reporting data on BIA; and (iii) including as outcomes body composition parameters or parameters associated with body water (total, extracellular) that can be estimated with BIA. Studies were excluded if they: (i) did not include humans; (ii) were conference abstracts; or (iii) were written in languages other than English.

### Data extraction

Two independent investigators (NV and CM) extracted key data from the included articles in a standardized Excel spread sheet and a third independent investigator (LS) checked these data, if needed. For each article, data were extracted on authors names, year of publication, country/continent, mean age, percentage of females, setting/main condition of inclusion, type of BIA, reference methods.

### Outcomes and statistical analysis

The outcomes of our investigation were: (i) body composition parameters in terms of fat-free mass, fat mass, and their derivates;( ii) hydration status in terms of total body water and its derivates. If available, data regarding sarcopenia and dehydration were extracted. We reported the data regarding BIA and the outcomes of interest narratively.

### Quality assessment

Two authors (NV, CM) independently carried out assessment of study quality using the Newcastle–Ottawa Scale (NOS) [25, 26]. The NOS ranks the quality of a case–control study, based on cases and controls definition, comparability between cases and controls, ascertainment of exposure, and non-response rate.

## Results

### Search results

**Fig. 1:**
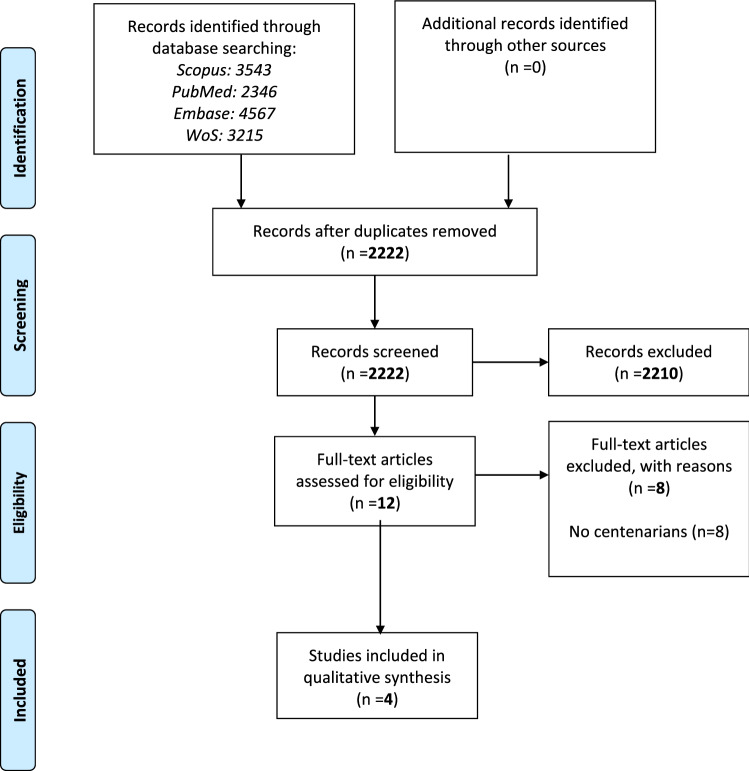
PRISMA flow-chart

A total of 2346 papers were screened and the full texts of twelve articles were finally retained. Among 2222 articles excluded at title/abstract screen, the main reasons for exclusion at this stage were that studies did not include centenarians or BIA was not used. Of the twelve full texts retained, four papers [27–30], giving information on three cohorts, were eligible. All the search results are shown in a PRISMA flow-chart (Fig. 1).

### Study and patient characteristics

The main descriptive findings of the included studies are reported in Table 1. The three cohorts involved a total of 291 centenarians (mean age: 100.5 ± 2.0 years) who were mostly women. Two studies were carried out in Europe and one in South America. The type of BIA used was different in all three studies. Two cohorts compared BIA to anthropometric measures and the other compared BIA to deuterium oxide dilution analysis. The quality of the included studies, according to the NOS, was generally low, particularly regarding cases and controls definition and how they were matched.

**Table 1:** General characteristics of the studies included

Author, year	Country	Condition	Sample size	Mean age (SD)	Males/females	Type of BIA	Reference tool
Duarte et al., 2019	Brazil	Community	24	101.1 (2)	0/24	MODEL 450	Deuterium oxide
da Silva et al., 2017 and da Silva et al., 2016	Portugal	Healthy	253	100.3 (2)	56/197	TANITA BC-420MA	Anthropometric measurements
Paolisso et al., 1996	Italy	Healthy	14	102.0 (0.8)	6/8	BIA 101/SC AKERN	Anthropometric measurements
Total			291	100.5 (2.0)	62/229		

*BIA*, bioelectrical impedance analysis; *SD*, standard deviation

### Main findings

The main findings of the BIA in centenarians are reported in Table 2. In one study, including 24 centenarians, bioelectrical impedance was compared against deuterium oxide dilution [27]. In this study, the authors found a statistically significant linear correlations between fat mass and fat-free mass estimated with both, BIA and deuterium oxide dilution. However, BIA overestimated fat-free mass and underestimated fat mass when compared to deuterium oxide dilution analysis.

**Table 2:** Main findings of the studies included

Author, year	Aim	Association with fat mass	Association with fat- free mass	Prevalence sarcopenia %	Prevalence of dehydration	Main findings
Duarte, 2019	To evaluate common body composition tools, such as anthropometry and BIA, and compare it with deuterium oxide in centenarian women	Lin's coefficient = 0.70	Lin's coefficient = 0.46	NA	NA	BIA overestimates FFM and underestimates FM, when compared to oxide deuterium
da Silva, 2017 and da Silva, 2016	To assess body composition, nutritional status and its differences between genders in a sample of Portuguese centenarians	According to body fat mass criteria assessed by BIA, the prevalence of obesity in study population was 6.0%, higher using anthropometric parameters. Significant correlation between fat mass assessed with bioelectrical impedance and waist circumference (*r* = 0.652, *p* < 0.001) and BMI (*r* = 0.793 *p* < 0.001)	NA	67.7	12.8	The risk for sarcopenia was higher in women, in those underweight and in osteoporotic individuals The amount of fat mass assessed with bioelectrical impedance was lower than their other anthropometric measures
Paolisso, 1996	To investigates glucose tolerance and insulin action in centenarians, including assessment of body composition with BIA	NA	*R* = 0.92, *p* < 0.001	NA	NA	A significant correlation between fat-free mass calculated by BIA and derived anthropometric measurements was found

*BIA*, bioelectrical impedance analysis, *BIA*, body mass index, *FFM*, fat-free mass; *FM*, fat mass; *NA*, not available

Similarly, Paolisso et al. found a strong linear correlation between BIA and fat-free mass evaluated using anthropometric tools in a small study carried out in Italy [29].

Finally, the largest cohort available was utilized for two studies [28, 30], showing that BIA had a significant agreement with anthropometric measures of fat mass in this population of centenarians. However, the proportion estimated to be obese using BIA, was only 6.0%, while this figure using anthropometric equations (Deurenberg and Gallagher’s equation) was significantly higher (77.7% and 42.0%, respectively). This is the only study reporting data regarding two conditions that might be diagnosed using BIA, i.e., sarcopenia and dehydration. Using the BIA, the first condition was defined as a fat-free mass value below 16.7 kg/m^2^, the latter as total body water below 45% in women and lower than 50% in men [28, 30]. In centenarians, a prevalence of sarcopenia of 67.7% was observed using a definition including only fat-free mass, while the prevalence of dehydration was 12.8%. Overall, these studies reported important information regarding sarcopenia and its risk factors (Table 2), and the association between BIA and fat mass.

## Discussion

In the present systematic review including four studies and 291 centenarians, we found that BIA was used for assessing body composition and hydration status in this particular population. However, most of the scientific literature available compared BIA with anthropometric measurements and not with other tools that can assess body composition and are gold standards for this purpose, e.g., Dual X-ray Absorptiometry (DXA).

The methods to assess body composition in older adults comprise the use of equations based on anthropometric and demographic parameters and in particular, to evaluate fat mass, such as the Deurenberg’s and the Gallagher’s formulas [30]. However, it should be acknowledged that age and BMI are parameters included in both formulas and are fundamental to calculate fat mass for a clinical approach in adults, while in older adults there are some limitations for these two parameters [30]. Centenarians included in the scientific available literature are usually lean, which may suggest that low fat mass could be considered as a favorable factor for longevity. However, it is widely known that low body weight and low BMI are important consequences of malnutrition in older adults [31]. Furthermore, high BMI values seem not associated with an increased risk of mortality in this population [32]. For all these reasons, the optimal range of BMI proposed for adults may not be appropriate for older adults and particularly for centenarians. Thus, the Deuremberg and Gallagher formulas seem poorly applicable in centenarians. Of importance, in the present review we did not find any anthropometric equation suitable for centenarians, since no study explored the accuracy of these equations by comparing them with a gold standard method, such as DXA.

One of the studies included [27] compared deuterium oxide dilution with anthropometric measurements and BIA to evaluate the most commonly used body composition tools in clinical practice. Deuterium oxide dilution is the gold standard for the determination of total body water (TBW) with high precision [27].

However, using deuterium oxide dilution, both fat-free mass and fat mass are indirect measures, since they are calculated from the distribution of water in the different body compartments. Similarly, the estimation of body composition with BIA may be affected by nutritional status, particularly in aged persons. Indeed, this study including a great majority of lean women demonstrated that BIA tends to overestimate fat-free mass and to underestimate fat mass [27]. The other method used to determine body composition in the same study was skinfolds measurements by means of the Durnin and Womersley anthropometrical equations, which showed a moderate-to-strong agreement with deuterium oxide dilution. However, this is applicable mainly in non-centenarians. When deuterium oxide dilution analysis and BIA were applied to centenarian women, there was a better agreement in the assessment of fat mass than fat-free mass, whereas fat-free mass agreement was better when using anthropometrical equations, suggesting that the use of BIA to determine body composition in centenarians requires further study.

Hypohydration is a state of water deficit, in which TBW is < 45% in women and < 50% in men [33]. Research in the evaluation of TBW in centenarians is limited to a single study identified in the present review where the prevalence of hypohydration is reported (12.8%), which was higher in women compared to men [28, 30]. However, it is possible that there is a bias because the definition of the cut-off points to indicate dehydration do not take into consideration the size of the participants. Likewise, this study suggested that an appropriate hydration status is a protective factor against osteoporosis, proposing that an intact skeletal muscle-bone axis is necessary also in centenarians. Several studies in older adults have reported that declines in muscle mass, bone mass, and muscle strength and power were related to sarcopenia and osteoporosis, which are a major causes of frailty and increased risk for disability, falls and fractures. Therefore, within the present systematic review, it was indicated that these concepts could be applied to centenarians.

Finally, gender is a relevant factor in centenarians because women are generally leaner than men, but this may not only be due to physiological differences in fat and fat-free mass, but also owing to differences in the prevalence of dehydration [30]. Differences in total body water, anthropometrical measurements, muscle mass and bone mass among men and women can justify different clinical and nutritional approaches. One study [30] found a mismatch between the quantity of muscle mass measured by BIA (67.7%) and the quality of muscle mass assessed with physical performance tests: practically no participant in this population could be considered sarcopenic, according to physical performance or muscle strength parameters.

The findings of the present study must be interpreted within its limitations. First, only four studies with limited sample sizes and limited population representation were identified. This is probably due to the inherent difficulties in enrolling this population, such as the high prevalence of disability and comorbidities (e.g., dementia). Second, no study was identified comparing the use of BIA versus DXA or other validated methods (e.g., air displacement plethysmography or four compartment models) for evaluating body composition parameters; therefore, it is not certain if the equations used in young and older adults can be applied in centenarians. In this regard, only one study included in the present review [29] reported the equation used for estimating fat-free mass [34], while the other three did not. Indeed, the use of validated equations in older people is essential to ensure that results are reliable. For example, where the equation was not been reported it is not known whether the ratio used to calculate fat-free mass from total body water is correct.

In conclusion, this systematic review indicates that bioelectrical impedance is rarely used in centenarians for research purposes, identifying only four studies. The studies included suggest that BIA could be used to estimate body composition parameters among this population in a clinical context. In particular, since BIA has limited costs and is transportable, it could be the ideal tool for assessing body composition and hydration status in older people across different settings. However, more research regarding the accuracy of BIA in centenarians is still needed.

## Data Availability

Data available upon reasonable request to corresponding author.
